# Minimally invasive versus open talonavicular arthrodesis: comparable functional outcomes in a retrospective comparative cohort

**DOI:** 10.1007/s00402-026-06282-8

**Published:** 2026-03-28

**Authors:** Felix Werneburg, Dariusch Arbab, Alexander Zeh, Karl-Stefan Delank, Natalia Gutteck

**Affiliations:** 1 Department of Orthopedic and Trauma Surgery, Martin Luther University Halle-Wittenberg, Halle, Germany; 2 Witten/Herdecke University, Witten, Germany

**Keywords:** Talonavicular arthrodesis, Minimally invasive surgery, Open technique, AOFAS, Functional outcomes, Complications, Operative time

## Abstract

**Background:**

Talonavicular (TN) arthrodesis is an established procedure for symptomatic TN joint pathology and medial column dysfunction. Open techniques provide direct visualization but may be associated with approach-related soft-tissue morbidity. Minimally invasive surgery (MIS) has been proposed to reduce tissue disruption; however, comparative clinical evidence focusing on functional outcomes remains limited. This study compared functional outcomes between MIS and open TN arthrodesis.

**Methods:**

This retrospective cohort study included 56 feet (55 patients) treated with TN arthrodesis between January 2021 and January 2025. Thirty-two feet underwent MIS TN arthrodesis and 24 feet an open approach. The primary endpoint was the American Orthopaedic Foot & Ankle Society (AOFAS) ankle–hindfoot score assessed preoperatively and at final follow-up. Secondary endpoints were operative time and complications. Radiographic follow-up was performed as part of routine care to assess osseous fusion and to guide postoperative rehabilitation.

**Results:**

Mean follow-up was 361 ± 69 days. Functional outcomes improved substantially in both groups. Mean AOFAS increased from 53.2 ± 17.6 to 89.4 ± 9.5 in the MIS group (mean gain 36.2 ± 13.3) and from 56.9 ± 14.5 to 87.7 ± 12.7 in the open group (mean gain 30.9 ± 9.1). Between-group differences were not significant for postoperative AOFAS (*p* = 0.853; Cohen’s d = 0.15) or operative time (59.2 ± 35.6 vs. 69.6 ± 35.1 min; *p* = 0.415). No complications were documented in the MIS cohort; one patient in the open cohort developed symptomatic nonunion requiring revision (between-group comparison *p* = 0.389).

**Conclusion:**

In this retrospective comparative cohort, MIS and open TN arthrodesis were associated with substantial and comparable functional improvement at short-to-midterm follow-up. Complications were uncommon overall; the low event rate precludes firm conclusions regarding comparative safety or nonunion risk. MIS may be considered when soft-tissue preservation is prioritized, and future prospective studies with standardized protocols and PROM-based outcomes are needed.

**Level of evidence:**

III.

## Introduction

The talonavicular (TN) joint is a biomechanical keystone of the medial column and a principal determinant of hindfoot–midfoot coupling. Degenerative or post-traumatic TN pathology typically results in medial midfoot pain, impaired push-off, and functional limitation—often exacerbated on uneven terrain—frequently prompting operative management once non-operative care fails. TN arthrodesis is an established option for isolated TN arthritis and medial-column pathology and may also be incorporated into broader reconstructive strategies in selected deformity patterns such as flexible progressive collapsing foot deformity (PCFD) [1–4]. 

Within the PCFD spectrum, consensus work has refined nomenclature and staging, emphasizing that procedures should be tailored to the dominant pathoanatomy and the clinically most relevant pain generator(s) [5]. Selective TN arthrodesis has therefore been explored as a targeted procedure in appropriately selected flexible PCFD cohorts, with reported improvements in functional outcomes [6–8]. 

Open TN arthrodesis via dorsomedial or medial approaches provides direct visualization and broad access for joint preparation and fixation but necessitates soft-tissue dissection in an anatomic region with limited envelope tolerance. Anatomical and cadaveric studies emphasize that access and joint preparation can vary between approaches and may influence technical execution and risk to surrounding structures [9, 10]. In parallel, minimally invasive (MIS) concepts have expanded across foot and ankle arthrodesis, driven by the hypothesis that reduced soft-tissue disruption may translate into lower approach-related morbidity; supportive comparative evidence exists in ankle arthrodesis [11, 12]. 

MIS TN arthrodesis typically relies on fluoroscopy-guided percutaneous joint preparation using a burr and percutaneous screw fixation. Early clinical series and technical reports suggest meaningful symptom relief and functional improvement after minimally invasive TN fusion, but comparative evidence with a primary focus on functional outcomes remains limited [13, 14]. Accordingly, the purpose of this study was to compare functional outcomes after MIS versus open TN arthrodesis using the AOFAS ankle–hindfoot score as the primary endpoint. Operative time and complications were reported as secondary endpoints.

## Materials and methods

### Study design, setting, and reporting standard

This retrospective comparative cohort study included consecutive patients treated with TN arthrodesis at a single tertiary academic center between January 2021 and January 2025. The manuscript is reported in accordance with STROBE recommendations for observational studies where applicable.

### Eligibility criteria

Patients were eligible if they had symptomatic TN-joint pathology (primary degenerative TN arthritis, post-traumatic TN arthritis, navicular osteonecrosis, flexible PCFD with TN-dominant symptoms, or other TN-centered indications) and had failed at least six months of non-operative treatment. Exclusion criteria were prior ipsilateral foot surgery and incomplete clinical documentation precluding assessment of the primary endpoint at final follow-up.

### Approach selection considerations

The surgical approach (MIS vs. open) was selected in routine clinical practice by the treating surgeon based on patient- and case-specific considerations. MIS was preferentially considered when soft-tissue preservation was prioritized and when the planned procedure was feasible through a percutaneous technique. An open approach was preferred when more extensive exposure was anticipated to be necessary, including severe deformity that may require broader correction, substantial osteophytes or joint collapse requiring direct visualization, or when additional procedures requiring open exposure were planned. These considerations reflect routine decision-making rather than predefined prospective inclusion/exclusion criteria.

### Exposure (surgical approach)

All patients underwent MIS or open talonavicular arthrodesis performed by a single senior fellowship-trained foot-and-ankle surgeon (N.G.) with extensive experience in fluoroscopy-guided minimally invasive hindfoot arthrodesis. The surgical approach (MIS vs. open) was selected based on surgeon preference, soft-tissue considerations, and feasibility in the given deformity and indication context (i.e., the ability to achieve the intended correction, joint preparation, and fixation through a percutaneous technique). Baseline characteristics and procedural heterogeneity (isolated TN fusion versus combined procedures) are reported to support interpretation of the comparative analyses.

## Surgical techniques

### MIS TN arthrodesis

**Fig. 1:**
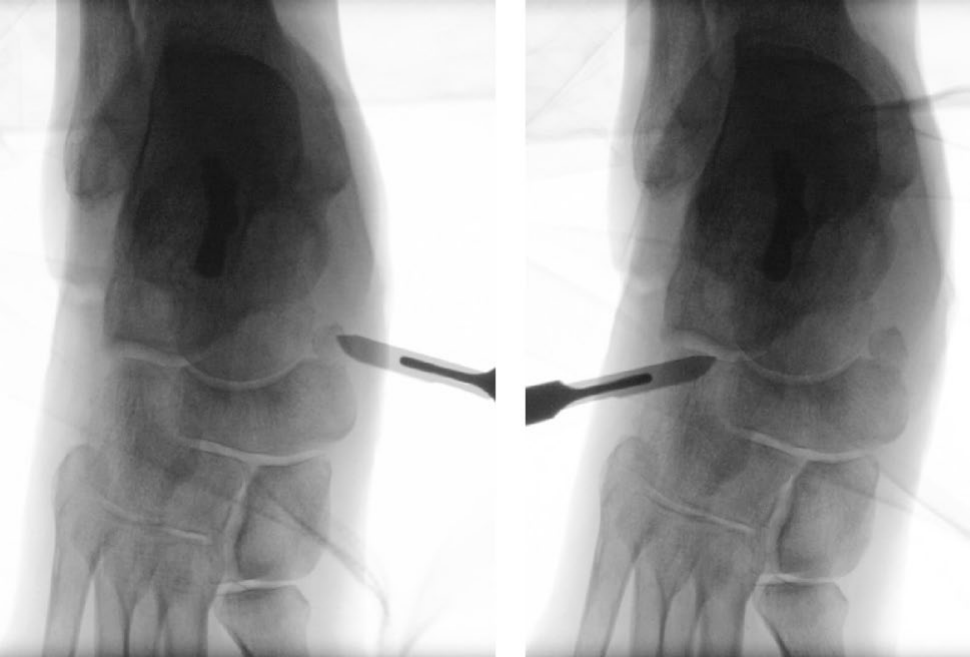
Intraoperative fluoroscopic views used to identify the TN joint line and plan percutaneous MIS portals prior to joint preparation: medial approach (left) and dorsolateral approach (right)

**Fig. 2:**
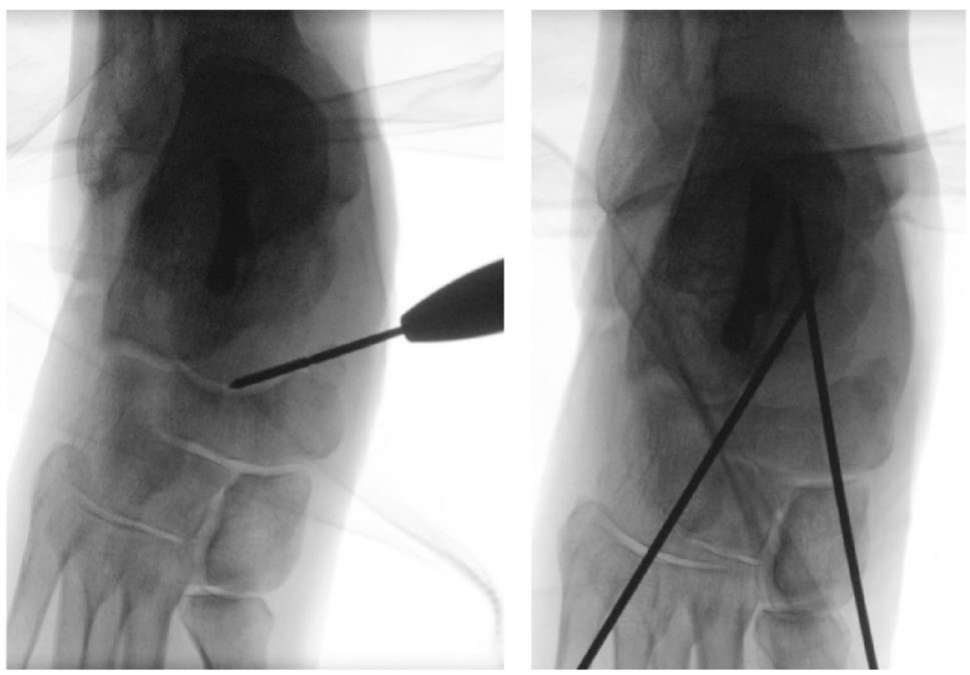
Intraoperative fluoroscopic confirmation of instrument position during MIS TN joint preparation (left) and temporary transfixation using percutaneous K-wires prior to definitive fixation (right)

MIS TN arthrodesis was performed through percutaneous medial and dorsolateral portals under fluoroscopic guidance. After patient positioning and sterile draping, the talonavicular joint line and planned portals were identified using orthogonal fluoroscopic views (Fig. 1). The medial portal was placed dorsally to the tibialis posterior tendon insertion; blunt dissection was used to minimize risk to neurovascular and tendon structures. Joint preparation was performed using a straight 20-mm Shannon burr introduced alternately through the medial and dorsolateral portals. Instrument position was verified repeatedly on fluoroscopy in two planes, and joint surface preparation was continued until a consistent loss of sharp subchondral contours suggested adequate decortication (Fig. 1). Microperforation (“microfracturing”) was then performed using the burr or a small drill to promote fusion. Bone debris was irrigated and removed, particularly from the subcutaneous tract. After correction to the intended alignment, temporary transfixation was achieved using percutaneous K-wires (Fig. 2). Definitive fixation was standardized with two cannulated compression screws (typically 6.0–6.5 mm) inserted in a crossed configuration to maximize interfragmentary compression and rotational stability. Final implant position and alignment were confirmed fluoroscopically. Bone grafting was not used in the MIS cohort. Safety considerations for percutaneous preparation and structures at risk have been described in cadaveric work and informed portal placement and trajectory discipline [15, 16]. 

## Open TN arthrodesis

Open TN arthrodesis was performed via a dorsomedial approach with direct joint exposure, thorough debridement/decortication, and correction of joint position prior to fixation. Fixation constructs varied: two cannulated compression screws in 16 feet, screw-plus-plate fixation in 4 feet, and staples in 4 feet. Bone graft (autologous or synthetic) was used selectively based on intraoperative judgment. Alternative fixation concepts (e.g., lateral fixation through a medial approach) have been described in the literature [17]. In cases requiring combined procedures, established technique concepts for medial-column/hindfoot arthrodeses were followed [18]. 

## Postoperative protocol and follow-up

**Fig. 3:**
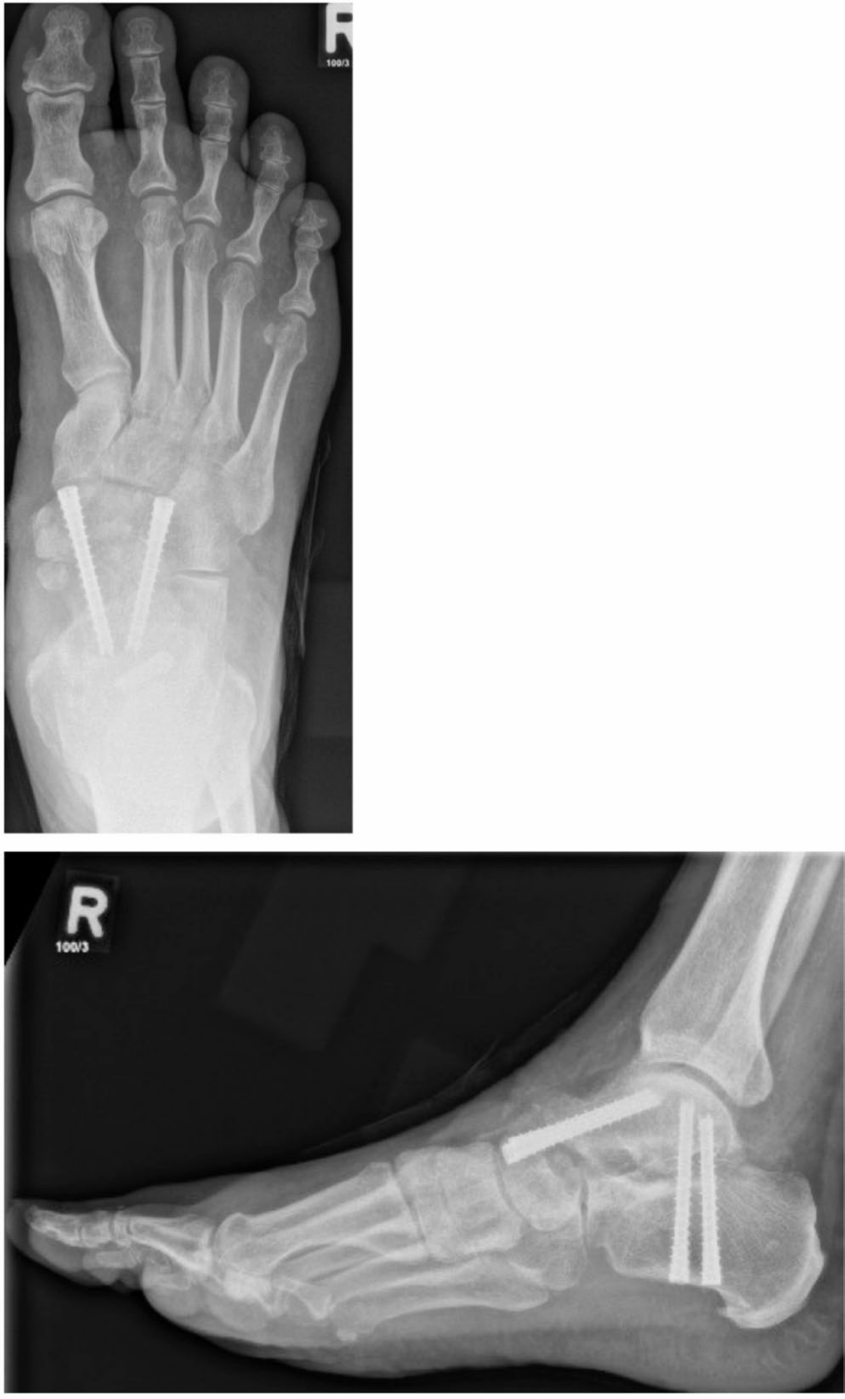
Postoperative radiographs after MIS TN arthrodesis: dorsoplantar projection (left) and lateral projection (right). In this case, a concomitant MIS subtalar arthrodesis was additionally performed

Postoperative management followed a standardized protocol: immobilization in a walker boot with touch-down weightbearing for six weeks, suture removal at approximately two weeks, progressive weightbearing beginning after six weeks if the clinical course was satisfactory, and transition to a stable shoe with a rigid sole from week eight onward. Routine clinical follow-up was scheduled at 6, 12, and 52 weeks. Standard-of-care radiographs were obtained at follow-up visits to assess osseous integration and implant position and to guide rehabilitation and weightbearing progression. Radiographs were assessed by the treating surgeon as part of routine follow-up; no independent blinded adjudication was performed. Radiographs were evaluated on orthogonal views (dorsoplantar and lateral; weight-bearing when tolerated). Osseous integration (radiographic fusion) was confirmed based on the following parameters on plain radiographs: (i) bridging trabeculation across the arthrodesis site with progressive obliteration of a distinct talonavicular joint line, (ii) absence of a progressive radiolucent gap at the fusion interface, and (iii) stable fixation without signs of hardware loosening, migration, or breakage. Computed tomography was not part of the routine postoperative protocol in asymptomatic patients and was reserved for clinically indicated cases (persistent symptoms and/or equivocal radiographs). Representative postoperative radiographs illustrating the typical implant configuration after minimally invasive talonavicular arthrodesis are provided in Fig. 3.

### Outcomes

#### Primary outcome: functional outcome

Functional outcome was assessed using the AOFAS ankle–hindfoot score, recorded preoperatively and at final follow-up. The AOFAS score comprises pain (40 points), function (50 points), and alignment (10 points), with higher scores indicating better status [19]. Recognized limitations of the AOFAS scoring system (mixed clinician-/patient-rated components, potential ceiling effects) are acknowledged [20–22]. 

#### Secondary outcomes

Secondary outcomes were:

1. operative time (minutes), and.
2. postoperative complications, defined a priori as wound problems, infection, thromboembolic events, neurovascular complications, symptomatic hardware issues requiring reoperation, and symptomatic nonunion requiring revision surgery. Complications were captured through chart review and follow-up documentation across the entire follow-up interval.

### Statistical analysis

Continuous variables were summarized as mean ± standard deviation; categorical variables as counts and percentages. The unit of analysis was the treated foot (56 feet in 55 patients). Distributional assumptions were assessed using visual inspection and Shapiro–Wilk testing; given the small sample size and to avoid reliance on normality assumptions, non-parametric tests were used for continuous variables. Between-group comparisons (MIS vs. open) were performed using the Mann–Whitney U test for continuous variables and Fisher’s exact test for categorical variables. Effect size for between-group differences in continuous outcomes was reported as Cohen’s d. All tests were two-sided, and statistical significance was set at *p* < 0.05. Because this was a retrospective consecutive cohort, no a priori sample size calculation was performed.

## Results

### Cohort characteristics

A total of 56 feet (55 patients) were included: 32 MIS and 24 open procedures. Mean follow-up was 356 ± 90 days in the MIS group and 367 ± 15 days in the open group.

**Fig. 4:**
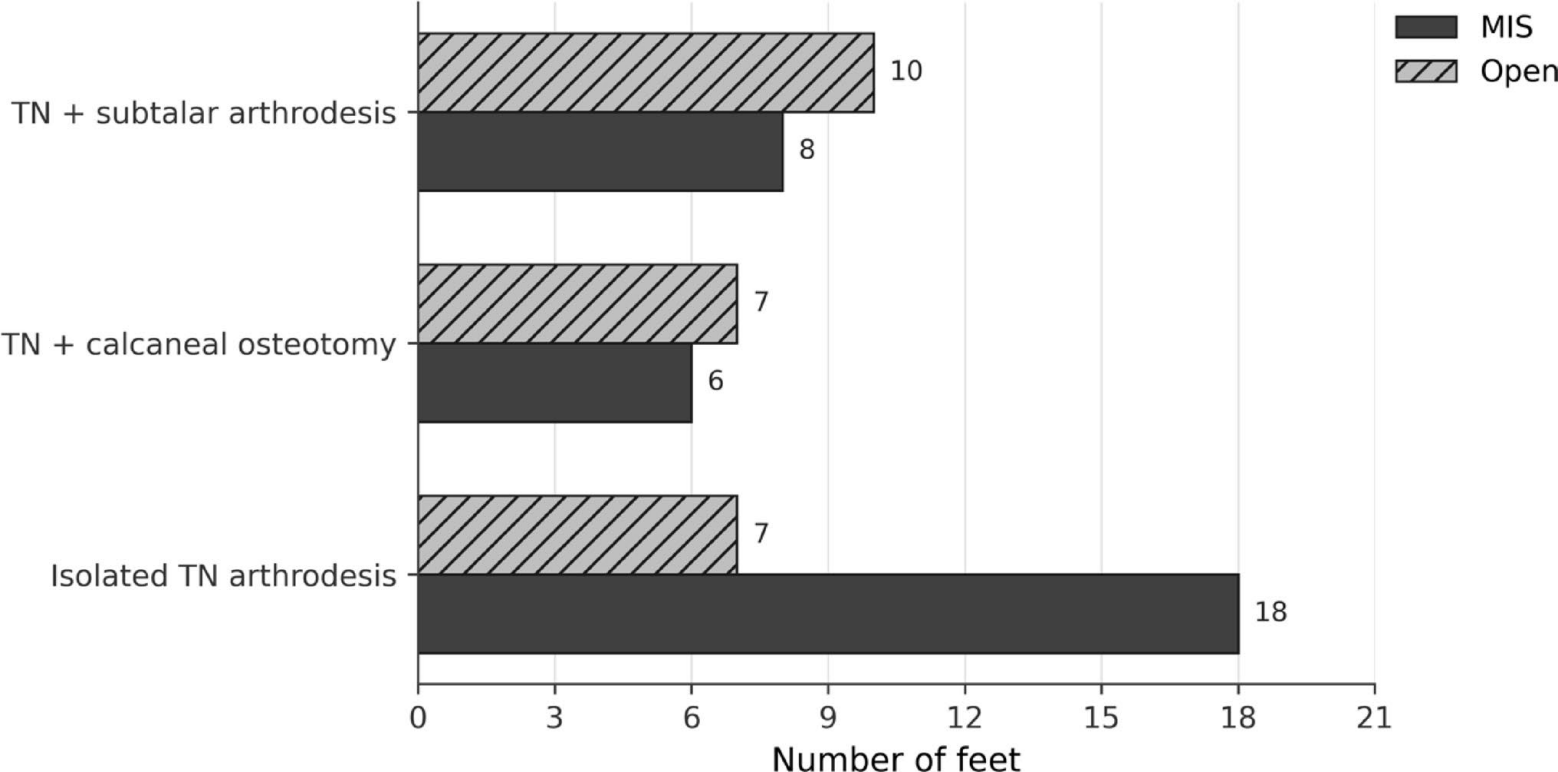
Procedure distribution by surgical approach. Horizontal bar chart showing the number of feet treated with (i) isolated talonavicular (TN) arthrodesis, (ii) TN arthrodesis combined with calcaneal osteotomy, and (iii) TN arthrodesis combined with subtalar arthrodesis. MIS = minimally invasive technique; Open = open technique

Table 1 summarizes baseline demographics, indication mix, comorbidities, and procedural heterogeneity. Baseline demographic characteristics did not differ significantly between groups (age: 54.8 ± 16.1 vs. 54.4 ± 19.5 years, *p* = 0.935; female sex: 62.5% vs. 66.7%, *p* = 0.785). Likewise, the prevalence of obesity (BMI ≥ 30 kg/m²) was not significantly different between cohorts (46.9% vs. 37.5%, *p* = 0.589). Isolated TN arthrodesis accounted for 56.2% of MIS cases and 29.2% of open cases, whereas combined procedures—particularly concomitant subtalar arthrodesis—were more frequent in the open group. The distribution of procedure types is visualized in Fig. 4.

**Table 1:** Patient demographics, comorbidities, procedure heterogeneity, and follow-up (Values are n (%) unless otherwise stated)

Variable	MIS (*n* = 32)	Open (*n* = 24)
Age (years), mean ± SD	54.8 ± 16.1	54.4 ± 19.5
Female sex	20 (62.5)	16 (66.7)
Side (left)	18 (56.3)	11 (45.8)
Diagnosis mix		
Primary (degenerative) TN arthritis	14 (43.8)	9 (37.5)
Posttraumatic TN arthritis	7 (21.9)	6 (25.0)
Navicular osteonecrosis	3 (9.4)	2 (8.3)
Flexible PCFD / adult-acquired flatfoot	6 (18.7)	6 (25.0)
Other indications	2 (6.1)	1 (4.2)
Comorbidities*		
Diabetes mellitus	7 (21.9)	4 (16.7)
Current smoker	8 (25.0)	4 (16.7)
BMI ≥ 30 kg/m²	15 (46.9)	9 (37.5)
Peripheral vascular disease	3 (9.4)	1 (4.2)
Rheumatoid arthritis	9 (28.1)	5 (20.8)
Procedural heterogeneity		
Isolated TN arthrodesis	18 (56.2)	7 (29.2)
TN arthrodesis + calcaneal osteotomy	6 (18.8)	7 (29.2)
TN arthrodesis + subtalar arthrodesis	8 (25.0)	10 (41.6)
Follow-up (days), mean ± SD	356 ± 90	367 ± 15

*Based on available chart documentation; denominators may vary

### Primary outcome: AOFAS ankle–hindfoot score

Functional outcomes improved markedly in both cohorts. Mean AOFAS increased from 53.2 ± 17.6 preoperatively to 89.4 ± 9.5 postoperatively in the MIS group (mean gain 36.2 ± 13.3 points). In the open group, mean AOFAS improved from 56.9 ± 14.5 to 87.7 ± 12.7 (mean gain 30.9 ± 9.1 points). Postoperative AOFAS did not differ significantly between techniques (*p* = 0.853), and the between-group effect size was small (Cohen’s d = 0.15).

**Fig. 5:**
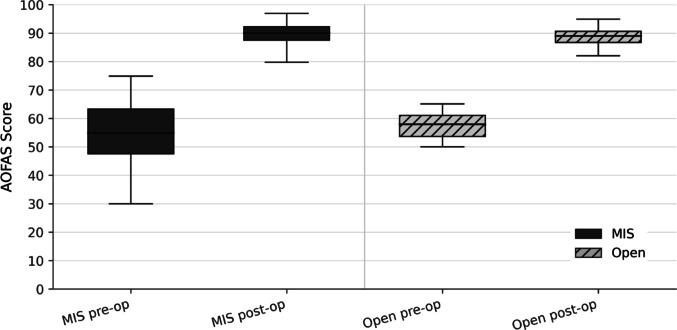
Functional outcomes (AOFAS score). Boxplots illustrating preoperative and final follow-up AOFAS scores for the MIS and open cohorts. Boxes represent the IQR with median; whiskers indicate the range within the dataset as displayed. MIS = minimally invasive technique; Open = open technique

**Fig. 6:**
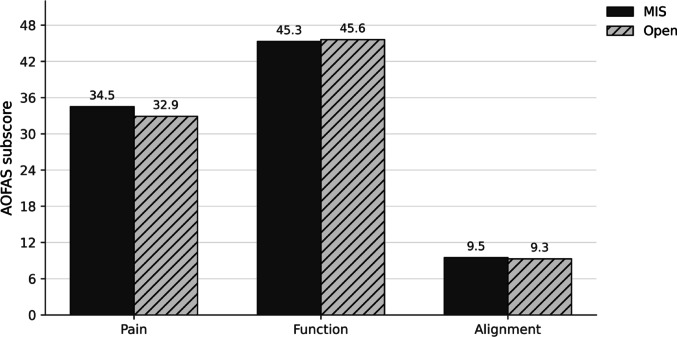
Postoperative AOFAS subdomains. Bar chart comparing mean postoperative AOFAS subscores (pain, function, and alignment) between MIS and open TN arthrodesis. Values above bars indicate group means. MIS = minimally invasive technique; Open = open technique

The distribution of pre- and postoperative scores is shown in Fig. 5, demonstrating a consistent shift toward high functional status in both groups. Subdomain analysis at final follow-up demonstrated no significant differences between techniques: pain (MIS 34.5 vs. open 32.9; *p* = 0.721), function (MIS 45.3 vs. open 45.6; *p* = 0.642), and alignment (MIS 9.5 vs. open 9.3; *p* = 0.803). Figure 6 summarizes these findings.

### Secondary outcomes: Operative time and complications

Mean operative time across the cohort was 63.2 ± 34.7 min. Mean operative time was 59.2 ± 35.6 min in the MIS group and 69.6 ± 35.1 min in the open group; this difference between groups was not statistically significant (*p* = 0.415).

No postoperative complications were recorded in the MIS cohort. One patient in the open cohort developed a symptomatic nonunion requiring revision surgery. No wound problems, infections, thromboembolic events, or sensory deficits were documented in either group. The difference in complication rates did not reach statistical significance (*p* = 0.389; Fisher’s exact test).

## Discussion

The principal finding of this study is that MIS TN arthrodesis was associated with substantial functional improvement comparable to an open approach at short-to-midterm follow-up, with no statistically significant intergroup differences in AOFAS outcomes. Operative time was comparable, and complications were infrequent overall.

### Functional outcomes in context of prior literature

TN arthrodesis is widely accepted as an effective intervention to relieve pain and improve function in TN-centered pathology, including degenerative and post-traumatic arthritis and deformity-related TN overload. Classical series and reviews describe reliable symptom improvement after isolated TN fusion, albeit with heterogeneity in indications and outcome instruments [2–4]. For hindfoot malalignment and combined fusion concepts, favorable clinical outcomes have likewise been reported when procedures are matched to deformity and pain generators [23]. 

Within PCFD, consensus work highlights heterogeneity of deformity mechanisms and reinforces individualized procedure selection [5]. Case series evaluating isolated TN arthrodesis in flexible deformity patterns have reported improvements in validated functional metrics, supporting that selective TN fusion can be reasonable in carefully selected patients [6–8]. Our cohort aligns with this clinically oriented literature by demonstrating substantial functional improvement irrespective of approach.

Recent comparative data from hindfoot arthrodesis literature further support this interpretation. Fiore et al. reported that both open and minimally invasive double and triple arthrodesis for rigid flatfoot deformity resulted in significant improvements in patient-reported outcomes, with no significant between-group differences in nonunion rates or time to union; however, wound dehiscence occurred more frequently after open surgery [24]. Our findings are broadly consistent with this central message, in that both surgical approaches were associated with substantial postoperative improvement and a low overall complication burden. At the same time, direct comparison should be made with caution, because the study by Fiore et al. used patient-reported instruments such as FFI, whereas the present cohort used the AOFAS ankle-hindfoot score as the primary outcome measure. Nevertheless, taken together, both studies suggest that minimally invasive arthrodesis techniques can achieve clinical improvement comparable to open surgery, while potentially offering advantages with respect to soft-tissue morbidity.

### Patient selection considerations

Although MIS TN arthrodesis offers potential advantages related to soft-tissue preservation, it may not be suitable for all surgical candidates. Based on our institutional practice, MIS was primarily used when percutaneous joint preparation and reliable screw fixation were feasible under fluoroscopy and when the deformity could be corrected without the need for extensive open release, debridement, or structural grafting. Conversely, an open approach may be more appropriate in cases requiring broad visualization, major deformity correction, revision surgery, compromised local biology, or when combined procedures necessitate open exposure. Importantly, approach allocation in this retrospective cohort was nonrandomized and guided by clinical judgment; therefore, patient selection is a potential source of confounding by indication and limits causal inference.

### MIS concepts and technical considerations

MIS TN arthrodesis has been described using minimal-incision/percutaneous strategies and arthroscopy-assisted approaches. A minimal-incision clinical series reported favorable clinical improvement with low soft-tissue morbidity, supporting the plausibility that reduced exposure can preserve the envelope without compromising clinical benefit [13]. Arthroscopic technique reports emphasize the potential for intra-articular visualization with limited soft-tissue disruption, but robust comparative clinical outcome data remain limited [14]. 

A key concern specific to percutaneous preparation is the proximity of neurovascular and tendon structures. Cadaveric studies delineate structures at risk during percutaneous TN preparation and underscore the importance of standardized portal placement, fluoroscopic control, and disciplined burr trajectory [15, 16]. These anatomical data provide a rationale for careful MIS execution and may contribute to low approach-related morbidity when the technique is performed in an experienced setting.

#### Operative time and clinical safety

Operative time did not differ statistically between approaches. This should be interpreted cautiously, as operative duration is influenced by concomitant procedures and construct selection. In this dataset, combined procedures were more common in the open group and fixation constructs were heterogeneous in the open cohort, whereas MIS fixation was standardized.

Complications were rare. The MIS cohort had no recorded complications, whereas one patient in the open cohort required revision for symptomatic nonunion. This low event rate limits inference but is consistent with the notion that TN arthrodesis can be challenged by nonunion risk depending on patient factors and construct mechanics.

### Fixation constructs and clinically relevant nonunion

Symptomatic nonunion requiring revision is a clinically meaningful endpoint. Fixation strategy has been examined in systematic reviews and cohort studies comparing screw-only with augmented constructs [25, 26]. Additional observational work has explored associations between hardware choice, patient factors, and nonunion risk [27, 28]. Our open cohort included variable constructs, reflecting real-world practice but limiting causal inference regarding construct choice versus approach. Future comparative studies should consider stratification by fixation construct and standardization of joint preparation.

#### Why functional outcomes should drive interpretation

The emphasis on clinical outcomes is patient-centered: pain relief, walking tolerance, and function are the most relevant endpoints for daily life. In our cohort, postoperative AOFAS scores approached the upper range in both groups, and subdomain analysis showed near-identical function and alignment subscores with no statistically significant differences in pain.

At the same time, the AOFAS score is a legacy instrument with well-described limitations, including mixed clinician- and patient-rated components and potential ceiling effects [20–22]. Future comparative studies should incorporate contemporary validated PROMs (e.g., FAAM, MOXFQ, PROMIS domains), which may improve sensitivity to differences at higher levels of postoperative function [22, 29]. 

#### Limitations

This study has limitations inherent to its design. First, treatment allocation was retrospective and nonrandomized, introducing potential selection bias and confounding by indication. Although baseline characteristics are reported, important factors relevant to approach selection could not be captured in a standardized manner for all patients, including objective deformity measures. Second, there was procedural heterogeneity, with combined procedures being more frequent in the open cohort, which may have influenced both functional outcomes and rehabilitation. Third, fixation strategies were heterogeneous in the open group compared with standardized fixation in the MIS group, limiting direct comparability of construct-related effects. Fourth, radiographic follow-up was performed as part of routine clinical care; CT imaging was not obtained routinely in asymptomatic patients and was reserved for clinical indications. Consequently, fusion assessment relied primarily on standard radiographs integrated with the clinical course, and time-to-radiographic union could not be determined uniformly beyond scheduled follow-up intervals. Fifth, adverse events were uncommon and the study was not powered to detect small between-group differences or rare complications, including nonunion, limiting inference regarding comparative safety. Sixth, follow-up was short-to-midterm and does not address long-term durability. Finally, outcomes relied on the AOFAS score as a legacy instrument with acknowledged limitations [20–22]. 

## Conclusion

In this retrospective comparative cohort, MIS and open TN arthrodesis produced substantial and comparable improvements in functional outcome at short-to-midterm follow-up. Operative time was similar, and complications were uncommon; the low event rate precludes firm conclusions regarding comparative safety or nonunion risk. MIS may be considered when soft-tissue preservation is prioritized, and future prospective studies with standardized protocols and PROM-based outcomes are needed.

## Data Availability

The datasets generated and/or analyzed during the current study are available from the corresponding author on reasonable request.
